# Improved Threshold Voltage Stability of p-GaN Gate HEMTs Under Off-State Drain Stress Using p-NiO RESURF Terminal

**DOI:** 10.3390/mi17040482

**Published:** 2026-04-16

**Authors:** Jun Pan, Xiangru Ye, Ruixi Jiang, Ailin Miao, Fuxiang Miao, Zhiyi Mao, Yanghu Peng, Hui Guo, Jianming Lei

**Affiliations:** 1 Zhongtian Broadband Technology Co., Ltd., Nantong 226463, China; 2 School of Electronic Science and Engineering, Nanjing University, Nanjing 210023, China; 3 School of Electrical Engineering, Nanjing Vocational University of Industry Technology, Nanjing 210023, China

**Keywords:** p-GaN gate AlGaN/GaN HEMT, Reduced surface field terminal, p-NiO, threshold voltage stability

## Abstract

A comparative study was undertaken to examine the V_TH_ stability of p-GaN gate high electron mobility transistors (HEMTs) without the p-NiO reduced surface field (RESURF) terminal and with the RESURF terminal under off-state drain voltage stress and negative gate stress, involving in-depth analyses of the net negative charge accumulation processes in the gate region and buffer layer, thereby revealing the degradation mechanisms of the devices. The findings indicate that the p-NiO RESURF terminal effectively enhances the stability of V_TH_ under off-state drain voltage stress by injecting holes into the buffer layer and hence initiating a light-pumping effect, and simultaneously also by flattening the electric field peak on the drain side beneath the gate and thus significantly mitigating hole loss in the gate region and electron capture in the buffer layer. This study provides a theoretical basis for the application of the p-NiO RESURF terminal in p-GaN HEMTs.

## 1. Introduction

GaN high electron mobility transistors (HEMTs) have attracted extensive attention in high-power switching applications due to their advantages such as high breakdown voltage, low on-resistance, and fast switching speed [1,2,3]. To simplify circuit design and enhance safety, normally-off operation has become a key requirement. Among various techniques for achieving enhancement mode, the p-GaN gate structure has become the mainstream choice for commercial devices [4,5]. As power switching devices, p-GaN gate HEMT devices are typically biased with a negative gate voltage in the off-state to prevent accidental turn-on, while the drain-source must withstand extremely high off-state voltages. This off-state condition, characterized by high drain voltage and negative gate voltage, leads to charge accumulation or loss in multiple regions such as the p-GaN layer, the AlGaN/passivation interface, and the buffer layer, causing severe reliability problems such as threshold voltage shift and on-resistance degradation [6,7,8,9]. To address this issue, researchers have proposed some terminal structures to improve the reliability of devices under off-state drain voltage stress. Wu et al., for example, employed a p-GaN active passivation (AP) layer on the drain side of the gate, which can effectively shield the surface traps from capturing electrons and significantly suppress the on-resistance degradation of p-GaN gate HEMT devices under off-state drain voltage stress [10,11]. However, the impact of this AP layer on threshold voltage shift has not been fully discussed. Additionally, the fabrication of the AP terminal structure requires multiple-step etching processes and strict control of etching depth.

NiO material is intrinsically p-type with a large bandgap, which can be fabricated by room-temperature magnetron sputtering without additional multi-step etching [12,13]. Moreover, the interface between the p-NiO and the AlGaN barrier layer shows a type-II band alignment [14,15], and thus holes may be injected into the buffer layer from the p-NiO under off-state drain voltage stress, which is expected to alleviate the electron trapping process in the buffer layer and thereby improve the threshold voltage stability of the device. A similar concept of utilizing favorable band alignment to enhance device performance has been demonstrated in AlGaN/GaN HEMTs by depositing an AlNO film, which offers competitive barrier heights for both electrons and holes and consequently suppresses gate leakage [16,17].

In this work, we introduce the p-NiO reduced surface field (RESURF) terminal structure into p-GaN gate HEMTs and study the impact of this terminal on the threshold voltage stability of the device under off-state drain voltage stress and the related mechanisms. Combining step-stress and gate current tests, we find that the p-NiO RESURF terminal can significantly suppress the hole loss in the gate region and the electron accumulation in the buffer layer, thereby significantly improving the threshold voltage stability of the device under off-state drain voltage stress.

## 2. Device Fabrication

**Figure 1:**
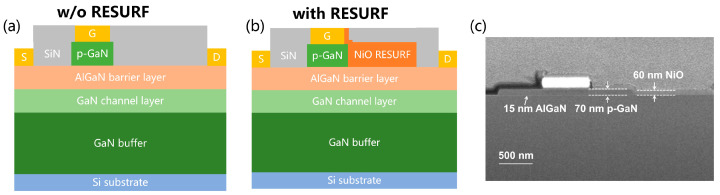
Schematics of GaN HEMT devices (**a**) w/o RESURF and (**b**) with RESURF terminal, and (**c**) SEM image of GaN HEMT with RESURF terminal.

The p-GaN gate AlGaN/GaN HEMT structure used in this work was grown by metal-organic chemical vapor deposition (MOCVD) on a Si (111) substrate, consisting of a 4-μm C-doped GaN buffer layer, a 300 nm undoped GaN channel, a 15 nm Al_0.25_Ga_0.75_N barrier, and a 70 nm p-GaN with a Mg concentration of ∼1 × 10^19^ cm^−3^. The device fabrication started with removing p-GaN outside the gate region by inductively coupled plasma (ICP) dry etching, and the source/drain ohmic contacts were then formed by electron-beam evaporation (EBE) of a Ti/Al/Ni/Au (30/150/50/100 nm) metal stack, followed by rapid thermal annealing at 850 °C for 30 s in N_2_ ambient. Subsequently, the Ni/Au (30/200 nm) stack was deposited on p-GaN as the Schottky gate contact by EBE. Then a 60 nm-thick and 2-μm-long p-NiO film with a hole concentration of 1 × 10^17^ cm^−3^ was deposited between the gate and drain by RF magnetron sputtering at room temperature, followed by a 100-nm-thick SiN_x_ passivation layer using plasma-enhanced chemical vapor deposition. Finally, a mesa isolation process was carried out by ICP dry etching, and the electrode window was opened by the lithography process. For reference, p-GaN gate HEMTs without the p-NiO RESURF terminal were also fabricated. The schematic cross-sectional structure of the two HEMTs with L_G_/L_GS_/L_GD_/W = 2/3/12/100 μm are displayed in Figure 1a and Figure 1b, respectively. The scanning electron microscope (SEM) image of the GaN HEMT with the RESURF terminal is shown in Figure 1c.

## 3. Results and Discussion

**Figure 2:**
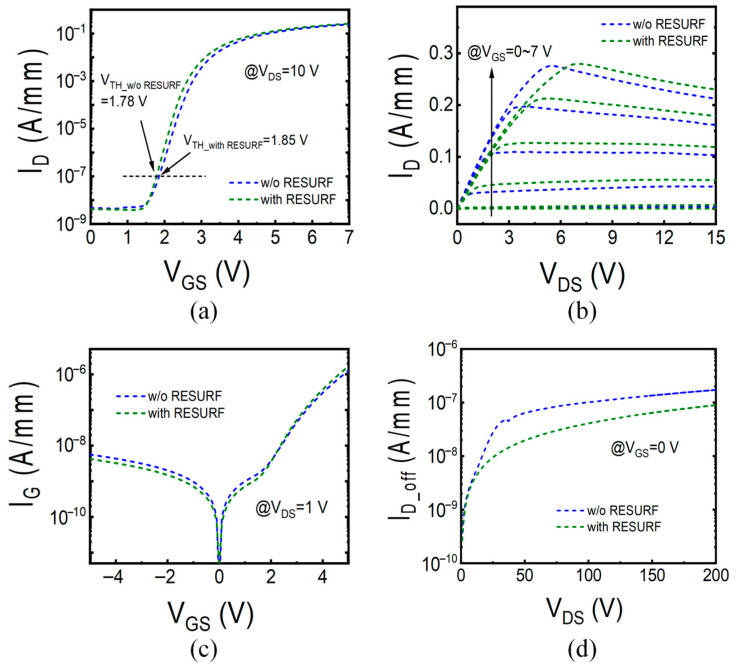
(**a**) Transfer and (**b**) output characteristics, and (**c**) gate current and (**d**) off-state drain leakage of HEMT devices with and without the p-NiO RESURF.

**Figure 3:**
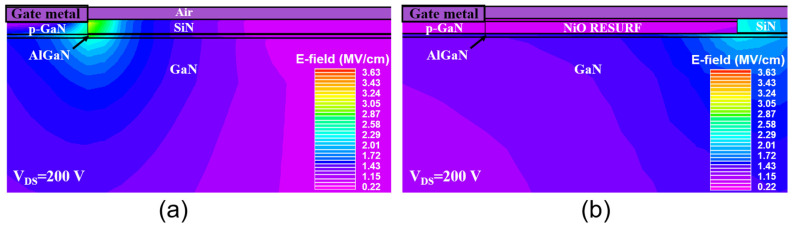
Electric field distributions of the HEMT devices (**a**) without and (**b**) with the RESURF terminal under V_DS_ = 200 V.

Figure 2a presents the transfer characteristics of two types of devices with and without the p-NiO RESURF terminal, measured under the condition of drain-source voltage V_DS_ = 10 V and gate-source voltage V_GS_ swept from 0 V to 7 V. The V_GS_ corresponding to a drain current I_D_ of 100 nA/mm is defined as the threshold voltage V_TH_. The V_TH_ for the device without RESURF is 1.78 V, while for the device with RESURF it is 1.85 V. Figure 2b illustrates the output characteristics of both devices with V_GS_ from 0 V to 7 V, and both devices exhibit the same saturation output current of 0.27 A/mm at V_GS_ = 7 V. Figure 2c shows the gate current I_G_ for both devices, measured with V_DS_ set at 1 V. Figure 2d depicts the off-state drain leakage current (I_D_off_) for both devices, measured with V_GS_ set at 0 V to ensure that the channel is turned off, and V_DS_ is swept from 0 V to 200 V. It can be observed that the I_D_off_ of the device with RESURF is lower than that of the device without RESURF, which can be attributed to the flattening of the electric field peak on the drain side beneath the gate by the p-NiO RESURF terminal, thereby reducing leakage through the buffer layer [18]. The flattening of the electric field peak on the drain side beneath the gate can be confirmed by simulating and comparing the electric field distributions of two HEMT devices under V_DS_ = 200 V, as shown in Figure 3a,b. Overall, the transfer characteristics, output characteristics, and gate currents of both devices are very similar. Therefore, it can be inferred that the p-NiO RESURF terminal has less impact on the on-state performances of the p-GaN gate HEMT devices.

**Figure 4:**
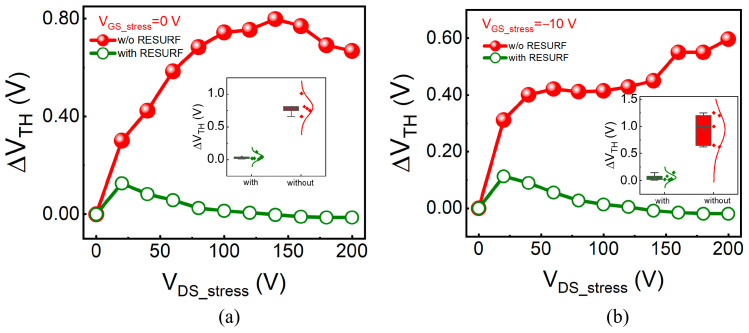
The trends of V_TH_ variation of HEMT devices with and without the p-NiO RESURF under drain bias stress at V_GS_stress_ of (**a**) 0 V and (**b**) −10 V, respectively. Inset: Statistical V_TH_ shift data.

To investigate the trends of V_TH_ variation under drain bias stress, we conducted step drain voltage stress (V_DS_stress_) tests. During the stress process, the gate voltage stress (V_GS_stress_) was fixed, while the V_DS_stress_ was gradually increased from 0 V to 200 V in steps of 20 V, with each step maintained for 100 s. Transfer characteristic measurements were performed before and between step-stress voltages to monitor the changes of V_TH_ in response to V_DS_stress_. The V_TH_ shifts (ΔV_TH_) at V_GS_stress_ = 0 V and −10 V with the substrate grounded (GND) are extracted and shown in Figure 4a and Figure 4b respectively with the error bars in the insets. Overall, the ΔV_TH_ of the device with RESURF is significantly smaller than that of the device without RESURF, and the trends in ΔV_TH_ are notably different. For the device without RESURF, the ΔV_TH_ continuously increases with the increase in V_DS_stress_, ultimately reaching saturation, after which it exhibits a slight decrease or increase. This behavior is attributed to the fact that under higher V_DS_stress_, holes in the p-NiO terminal can inject into the buffer layer more easily, thus resulting in a light-pumping effect, which denotes the process wherein light emitted from the recombination of electrons and holes in the buffer layer excites electrons confined in trap states. This light-pumping effect will significantly suppress the electron capture process and prevent ΔV_TH_ from further increasing or even decreasing. In contrast, for the device with RESURF, the ΔV_TH_ reaches a maximum value at V_DS_stress_ = 20 V and subsequently decreases with increasing V_DS_stress_. The positive shift in V_TH_ indicates the accumulation of net negative charge in the gate region or buffer layer, and the significant difference in ΔV_TH_ between the two devices suggests that the p-NiO RESURF terminal suppresses the accumulation of net negative charge, thereby significantly enhancing the stability of V_TH_ under off-state leakage voltage stress. In addition, the p-NiO/AlGaN interface may present oxidation or diffusion processes under high off-state stress, which also has an important effect on V_TH_ stability by changing interface fixed charges, as reported by Shen et al. [19]. However, oxidation or O atom diffusion at the p-NiO/AlGaN interface will weaken the effect of hole injection on the V_TH_ shift due to a higher potential barrier of Al_2_O_3_ at the oxidized interface of the p-NiO/AlGaN or reduced hole concentration in the p-NiO bulk resulting from O atom diffusion, and this phenomenon did not occur in the ΔV_TH_ variation trend with V_DS_stress_, as presented in Figure 4.

**Figure 5:**
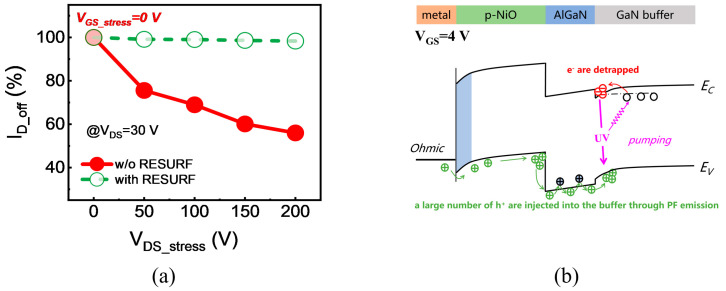
(**a**) Variations of I_D_off_ with V_DS_stress_ for HEMT devices with and without the p-NiO RESURF at V_GS_stress_ = 0 V and V_DS_ = 30 V, and (**b**) band diagram of the p-NiO/AlGaN/GaN buffer.

To verify the accumulation of net negative charge in the gate region or buffer layer, we tested the variation of I_D_off_ with respect to V_DS_stress_ for both devices. During the tests, V_GS_stress_ was set to 0 V, and V_DS_stress_ was gradually increased to 200 V. As shown in Figure 5a, after stress, the I_D_off_ of the device without RESURF exhibits a significant decrease, while that of the device with RESURF shows only a slight reduction measured at V_DS_ = 30 V. It is well known that traps within the buffer layer can capture electrons and form a barrier, impeding the flow of electrons from the source to the drain and resulting in a decrease in I_D_off_ [20,21]. Therefore, the decrease in I_D_off_ indicates that the electron capture process has occurred within the buffer layer under stress. As shown in the band diagram of Figure 5b, under the vertically downward electric field produced by high drain stress voltage, holes from the p-NiO terminal will cross the AlGaN barrier layer into the GaN buffer layer, generating a light-pumping effect, thereby achieving the effect of electron de-trapping [22,23]. In addition, the injected holes may directly recombine with trapped charges in defect states. Owing to these mechanisms, the rise in I_D_off_ is suppressed, and the V_TH_ stability is substantially improved.

**Figure 6:**
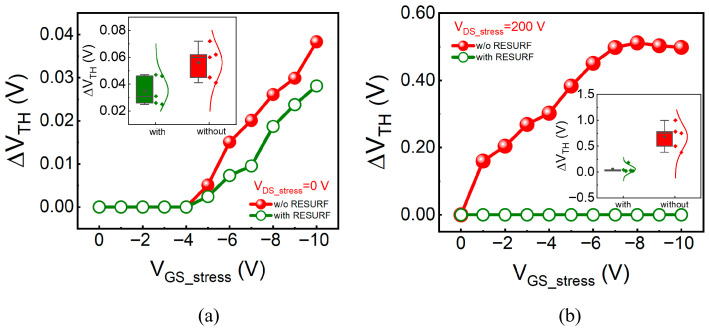
V_TH_ variations of HEMT devices with and without the p-NiO RESURF under negative gate voltage at V_DS_stress_ of (**a**) 0 V and (**b**) 200 V, respectively. Inset: Statistical V_TH_ shift data.

Further analysis of the impact of negative gate voltage on V_TH_ stability was conducted by step-stress measurements. During tests, V_DS_stress_ was fixed while V_GS_stress_ was gradually increased from 0 V to −10 V. Negative gate voltage stress could also induce net charge accumulation and modulate the impact ionization process induced by high drain voltage stress. To clarify the specific effect mechanisms, tests were performed under two conditions: V_DS_stress_ = 0 V and 200 V. At V_DS_stress_ = 0 V, the degradation of the device is solely attributed to negative gate voltage, eliminating the influence of charge accumulation caused by high drain voltage. The shifts in V_TH_ for both devices at V_DS_stress_ = 0 V are illustrated in Figure 6a with the error bars in the inset. Overall, both devices exhibit a slight positive shift in V_TH_, indicating that negative gate voltage can also lead to minor negative charge accumulation. The ΔV_TH_ of the device with RESURF is slightly less than that of the device without RESURF, suggesting that the p-NiO RESURF terminal also exerts some regulatory effect on the negative charge accumulation caused by negative gate voltage. At V_DS_stress_ = 200 V, the shifts in V_TH_ are depicted in Figure 6b with the error bars in the inset. Overall, the ΔV_TH_ of the device with RESURF is significantly smaller than that of the device without RESURF. As V_GS_stress_ increases, the ΔV_TH_ of the device without RESURF first continuously increases and then gradually saturates, while the ΔV_TH_ of the device with RESURF shows less change. These results confirm that the net negative charge accumulation is primarily induced by the drain voltage. For the device without RESURF, negative gate voltage has a significant regulatory effect on the net negative charge accumulation or impact ionization process. In contrast, for the device with the p-NiO RESURF terminal, negative gate voltage has almost no effect on ΔV_TH_.

**Figure 7:**
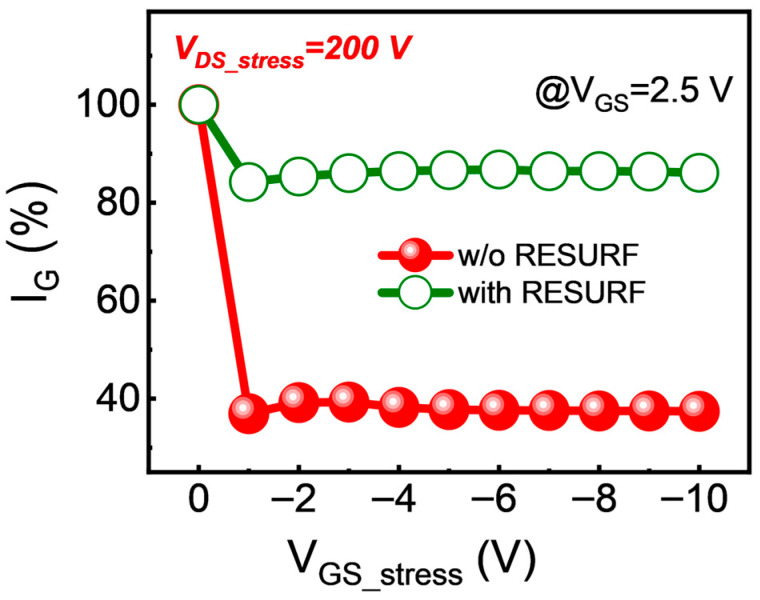
The variation of I_G_ with V_GS_stress_ for HEMT devices with and without the p-NiO RESURF at V_DS_stress_ = 200 V.

Finally, the effect of the p-NiO RESURF terminal on degradation of I_G_ with V_GS_stress_ under high drain voltage was investigated. The variation of I_G_ with V_GS_stress_ for both devices at V_DS_stress_ = 200 V is presented in Figure 7. Overall, both devices exhibit noticeable degradation in I_G_ at V_GS_stress_ = −1 V, followed by only slight fluctuations with increasing V_GS_stress_. Moreover, the degradation of I_G_ for the device with RESURF is significantly less than that for the device without RESURF. The increase in V_GS_stress_ not only suppresses the process of hole generation by impact ionization but also attracts more holes to the gate [24]. These two effects reach a balance with the increasing V_GS_stress_, and so the effect of increased V_GS_stress_ on hole loss in the gate region is almost negligible. Based on this phenomenon, it can be further speculated that the loss of holes in the gate region is primarily induced by high drain voltage. The main reason that the p-NiO RESURF can inhibit hole loss in the gate region is that this terminal can flatten the electric field beneath the gate.

Overall, for the device without RESURF, negative gate voltage under high drain voltage exerts a regulatory effect on the impact ionization at the drain side beneath the gate, consequently affecting the net negative charge accumulation in the buffer layer and gate region. In contrast, for the device with RESURF, the p-NiO terminal can effectively suppress the net negative charge accumulation in the buffer layer and gate region through the light-pumping effect and by flattening the electric field.

## 4. Summary

A comparative study has been conducted on the V_TH_ shifts of devices without RESURF versus those with RESURF under off-state drain voltage stress. Experimental results indicate that the V_TH_ shift of the device with RESURF is significantly smaller than that of the device without RESURF, which can be primarily attributed to: (1) the light-pumping effect induced by the p-NiO terminal by injecting holes into the buffer layer under the vertical electric field and significantly suppressing electron capture within the buffer layer; and (2) the flattening of the electric field peak on the drain side beneath the gate by using the p-NiO terminal and alleviating hole loss in the gate region. Furthermore, for the device without RESURF, negative gate voltage has a slight effect on the net negative charge accumulation in the buffer layer and gate region, consequently affecting the V_TH_ shift. In contrast, for the device with RESURF, this effect is almost negligible. These disparities further highlight the significant advantage of the p-NiO RESURF terminal in enhancing V_TH_ stability of p-GaN HEMTs.

## Data Availability

The original contributions presented in this study are included in the article. Further inquiries can be directed to the corresponding author.
